# Protocol for MR1-ligand cross-linking to discover metabolite antigens by proteomics

**DOI:** 10.1016/j.xpro.2026.104399

**Published:** 2026-03-02

**Authors:** Hannah Thomas, Garry Dolton, Lucia F. Cardo, Nicola Ternette, Andrew K. Sewell, Thierry Schmidlin

**Affiliations:** 1 Division of Infection and Immunity, Cardiff University School of Medicine, Cardiff CF14 4XN, UK; 2 University of Dundee, School of Life Sciences, Dow Street, Dundee, Scotland DD1 5EH, UK; 3 Systems Immunology Research Institute, Cardiff University, Cardiff, Wales, UK; 4 Division of Infection and Immunity, Kumamoto University, Kumamoto, Japan; 5 Institute of Immunology, University Medical Center of the Johannes Gutenberg-University Mainz, 55131 Mainz, Germany; 6 Research Center for Immunotherapy (FZI), University Medical Center of the Johannes-Gutenberg University, 55131 Mainz, Germany

**Keywords:** Health sciences, Immunology, Metabolism, Proteomics

## Abstract

Here, we present a protocol for the discovery of MR1-bound metabolite ligands by stabilizing Schiff base interactions between MR1 and its ligands. We describe steps for enriching MR1-ligand complexes using MR1-expressing A549 cells and covalently cross-linking ligands to the MR1 lysine 43 residue via reductive amination. We then detail procedures for identifying ligand-modified MR1 peptides by mass spectrometry and for further validation of candidate ligands using MR1 surface upregulation and TCR functional assays.

For complete details on the use and execution of this protocol, please refer to Schmidlin et al.^1^

## Highlights

• Protocol for MR1 ligand discovery by crosslinking and proteomics
• Affinity enrichment of MR1-ligand complexes from engineered cell lines
• Mass spectrometry-based detection of MR1-ligand adducts
• Integrated data analysis for ligand candidate selection and biochemical validation

## Abstract (editor highlights)

Publisher’s note: Undertaking any experimental protocol requires adherence to local institutional guidelines for laboratory safety and ethics.

## Graphical abstract

**Figure:**
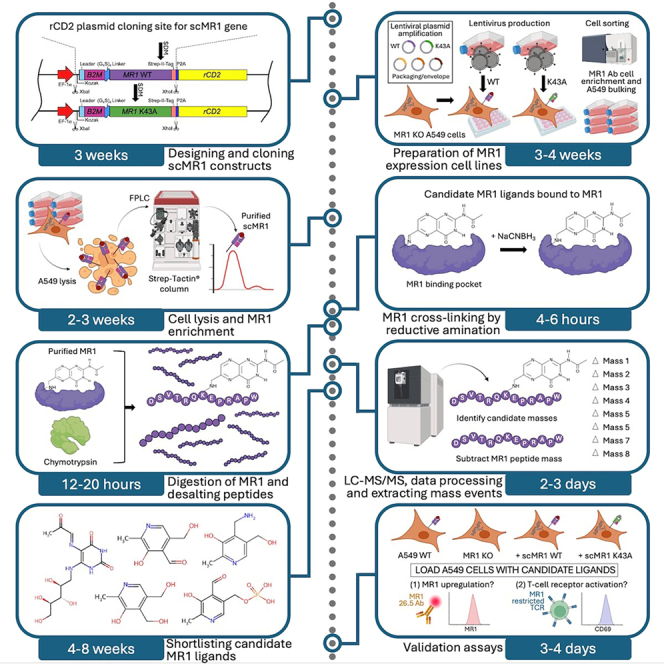

## Before you begin

### Background

Major histocompatibility complex (MHC) class I–related protein 1 (MR1) is an antigen-presenting molecule best known for presenting small-molecule metabolites derived from microbial and endogenous metabolic pathways in the context of bacterial infection and cancer.^2^ While the importance of endogenous ligand presentation is increasingly recognized, the full repertoire of MR1-associated metabolites remains incompletely defined. Some MR1 ligands can form a Schiff base with the lysine 43 residue of MR1 (K43),^3^ a mechanism that has been demonstrated for several vitamin B–derived metabolites that bind to MR1. In the protocol described here, this chemical feature is leveraged as an analytical handle to covalently stabilize ligands while bound in the MR1 binding groove, enabling direct antigen discovery using mass spectrometry–based proteomics.

**Figure 1:**
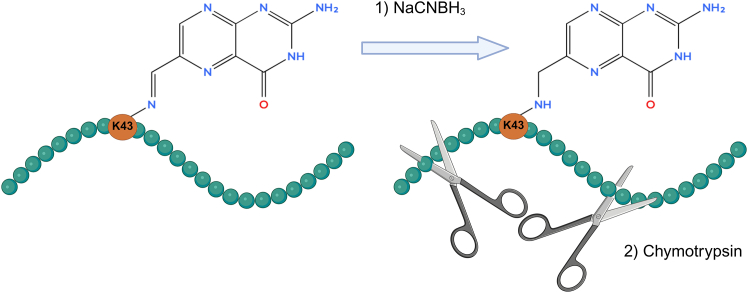
Workflow for stabilizing MR1–ligand Schiff bases and generating ligand-cross-linked MR1 peptides NaCNBH_3_ stabilizes the labile Schiff base between MR1 lysine 43 (K43) and the ligand by reductive amination forming a stably cross-linked MR1-ligand complex. Chymotryptic digestion is then used to generate the ligand modified MR1-peptide DSVTRQKEPRAPW, allowing to detect ligands as covalent adducts by LC-MS/MS-based proteomics.

Because Schiff base interactions are chemically labile, the MR1–ligand linkage is readily hydrolyzed once MR1 is denatured and loses its tertiary structure during sample processing. Consequently, conventional MR1 workflows typically rely on ligand dissociation, which results in a loss of information about the direct ligand–MR1 interaction. To preserve this interaction, the protocol employs reductive amination using sodium cyanoborohydride (NaCNBH_3_), which converts the unstable imine into a stable secondary amine.^4^^,^^5^^,^^6^^,^^7^ This stabilization allows K43-linked ligands to remain attached during proteolytic digestion, generating ligand adducts of the diagnostic MR1 peptide DSVTRQKEPRAPW (Figure 1). Subsequent Δ-mass analysis enables inference of ligand masses directly from the proteomic data and facilitates shortlisting of candidate ligands.

We first described this methodology in Schmidlin et al.,^1^ which enabled the identification of pyridoxal and pyridoxal 5′-phosphate (PLP) as MR1 ligands. In the protocol below, we describe this full workflow as used in our primary study, covering all steps from generating MR1-expressing cell lines to Δ-mass analysis, ligand prioritization, and biochemical validation. Although demonstrated in A549 cells, the approach is adaptable to additional model systems expressing MR1, provided that appropriate culture, enrichment, and safety approvals are in place. Prior to starting the protocol, ensure that all LC-MS instrumentation is fully calibrated, and that the required buffers, reducing agents, and affinity materials are freshly prepared according to the materials and equipment section.

### Innovation

This protocol introduces a dedicated workflow for the identification of MR1-bound metabolite ligands, an area where experimental tools have been limited so far. The key innovation of this protocol is the stabilization of Schiff base interactions between MR1 K43 and its small molecule ligands employing reductive amination. This reaction retains antigenic ligands in the MR1 binding groove by forming a stable covalent bond between metabolite and MR1 that is preserved during sample preparation, proteolytic digest and LC-MS-based proteomics allowing for reliable detection of ligand-modified MR1 peptides that would otherwise be lost during sample handling.

Downstream analysis incorporates Δ-mass screening and ligand shortlisting, providing a systematic approach to gain information on the molecular mass of known MR1 ligands and novel candidate compounds directly from the proteomics data. The workflow is further supported by orthogonal well-established validation assays towards candidate ligands, such as MR1 surface expression and MR1-restricted T cell activation via CD69 expression assays, which directly link mass spectrometry findings to functional antigen presentation and immunogenicity assessment.

Compared to previous approaches that relied on ligand pulsing or indirect reporter assays, this protocol provides a direct biochemical readout of MR1-ligand complexes with increased sensitivity. The combination of cross-link stabilization, optimized LC–MS/MS acquisition, and functional validation creates a versatile platform that can be applied to diverse cell systems and experimental conditions. Together, these innovations expand the toolkit for antigen discovery and enable deeper insights into the role of MR1 in immunometabolism.

### Preparation

#### Cell culture

**Timing: Variable**

This section describes the culture and expansion of all cell types required throughout the protocol. Each cell type serves a defined purpose in the ligand-cross-linking workflow: (1) HEK293T cells are used for lentiviral production of scMR1 constructs, (2) A549 MR1 KO cells are used to generate strep-tagged single chain MR1/β2M fusion protein (scMR1)-overexpressing lines (WT and K43A), which serve as the source material for MR1–ligand enrichment, and (3) A549 WT, A549 MR1 KO, and Jurkat Triple Parameter Reporter (TPR) cells are cultured for validation assays.

1. Culture adherent HEK293T cells for lentivirus production:
  a. Seed 0.5–1 × 10^6^ HEK293T cells in 12 mL D10 media in a T75 culture flask.
  b. Incubate at 37 °C in a 5% CO_2_ environment until confluency reaches >90% (typically within 4-5 days).
  c. Remove the R10 medium.
  d. Add 12 mL of 2 mM detachment medium to detach the cells from the culture flask.
  e. Incubate at 37 °C in a 5% CO_2_ environment for 5-10 minutes.
  f. Harvest the cells into a centrifuge tube.
  g. Centrifuge at 400 *× g.*
  h. Remove the supernatant.
  i. Replate cells back into the culture flask at a 1:5-1:10 ratio in 12 mL D10 media.
2. Culture adherent A549 MR1 KO + scMR1-WT, A549 MR1 KO + scMR1-K43A cells for cell lysis and MR1 enrichment
  a. Seed 1-2 × 10^6^ of A549 MR1 KO + scMR1-WT and A549 MR1 KO + scMR1-K43A cells in 25 mL R10 media in T175 culture flasks.
  b. Incubate at 37 °C in a 5% CO_2_ environment until confluency reaches >90% (typically 6-7 days).
  c. Remove the R10 medium.
  d. Add 25 mL of 2 mM detachment medium to detach the cells from the culture flask.
  e. Incubate at 37 °C in a 5% CO_2_ environment for 10-15 minutes.
  f. Harvest the cells into a centrifuge tube.
  g. Centrifuge at 400 *× g.*
  h. Remove the supernatant.
  i. Replate over 2-3 additional T175 flasks in 25 mL R10 media per flask.

***Note:*** The MR1-expressing A549 cell lines described here are generated during the step-by-step section at the end of Step 11 and will need to be cultured for several weeks to bulk up sufficient cell numbers for cell lysis and MR1 enrichment.

3. Culture A549 wildtype (WT), A549 MR1 KO (clone 9), A549 MR1 KO + scMR1-K43A cells for validation assays.
  a. Seed 0.5–1 × 10^6^ A549 wildtype (WT), A549 MR1 KO (clone 9), and A549 MR1 KO + scMR1-K43A cells in 12 mL R10 media in T75 culture flasks.
  b. Incubate at 37 °C in a 5% CO_2_ environment until confluency reaches >90% (typically within 4-5 days).
  c. Remove the R10 medium.
  d. Add 12 mL 2 mM detachment medium to detach the cells from the culture flask.
  e. Incubate at 37 °C in a 5% CO_2_ for 10-15 minutes.
  f. Harvest the cells into a centrifuge tube.
  g. Centrifuge at 400 *× g.*
  h. Remove the supernatant.
  i. Replate cells back into the culture flask at a 1:10-1:20 ratio of cells in 12 mL R10 media.
4. Culture TPR Jurkat cells (untransduced and MC.7.G5 TCR transduced) for validation assays.
  a. Seed 0.5–1 × 10^6^ of untransduced and MC.7.G5 TCR transduced TPR Jurkat cells in 20-30 mL R10 media in T75 culture flasks.
  b. Incubate at 37 °C in a 5% CO_2_ environment until confluency reaches >1 × 10^6^ cells/mL (typically within 5-6 days).
  c. Split cells by 1:10-1:20:
    i. Resuspend the culture.
    ii. Remove 90-95% of the volume into the waste.
    iii. Add a fresh replacement volume of R10 media.

***Note:*** TPR Jurkat cells are TCR KO CD8ab+ triple parameter reporter (TPR) cells. The MC.7.G5 TCR-transduced TPR Jurkat cells also have a rCD2 co-marker for TCR expression.

## Key resources table

**Table:** 

REAGENT or RESOURCE	SOURCE	IDENTIFIER
**Antibodies**		

Anti MR1-PE: clone 26.5 (2 μg/mL)	BioLegend	RRID:AB_2562969
Anti-rCD2: clone OX-34 PE conjugated (2 μg/mL)	BioLegend	RRID:AB_2073811
CD8 APC-Vio770: clone BW135/80 (1:100)	Miltenyi Biotec	RRID:AB_2725983
CD69 APC: clone FN50 (2 μg/mL)	BioLegend	RRID:AB_2922660

**Bacterial and virus strains**		

XL10-Gold Ultracompetent cells	Agilent	Cat# 200315
Third generation lentiviral transfer vector backbone pELNS: XbaI-Kozak-β2M-GS linker-MR1 (without leader sequence)-XhoI-P2A-rCD2-SalI-Stop	provided by Dr. James Riley, University of Pennsylvania, PA, USA	N/A

**Chemicals, peptides, and recombinant proteins**		

Carbenicillin disodium salt	ThermoFisher Scientific	Cat# 10396833
Agar bacteriological	Fisher Scientific	Cat # 10048991
Opti-MEM ™	ThermoFisher Scientific	Cat# 31985070
Polyethylenimine	Merck	Cat# 306185
Dulbecco modified Eagle-Medium (DMEM)	Merck	Cat# D6429
Fetal Bovine Serum (FBS)	Merck	Cat# F7524
Penicillin-Streptomycin	Merck	Cat# P4333
L-glutamine	Merck	Cat# G7513
Hexadimethrine bromide (polybrene®)	Santa Cruz	Cat# sc-134220
Roswell Park Memorial Institute 1640 medium (RPMI 1640)	Merck	Cat# R8759
Dulbecco’s Phosphate Buffered Saline (D-PBS)	Merck	Cat# D8537
EDTA	Merck	Cat# 6758
Formaldehyde	Fisher Scientific	Cat# 10160052
Acetyl-6-formylpterin	Cayman Chemicals Schircks Laboratories	Item No. 23303 Product number 11.418
DMSO	Thermo Scientific (Invitrogen™)	Cat# D12345
Sodium Chloride (NaCl)	Fisher Scientific	Cat# 10194763
Tris base	Thermo Scientific	Cat# BP152-500
NP-40	Thermo Scientific	Cat# PI85124
Streptavidin	Thermo Scientific	Cat# PI21122
10× Buffer W, Strep-Tactin XT Wash buffer	Stratech Scientific Ltd	Cat# 2-1003-100-iba-100ML
10× Buffer BXT, Strep-Tactin XT Elution Buffer	Stratech Scientific Ltd	Cat# 2-1042-025-IBA-25ML
Buffer XT-R; Regeneration Buffer	Stratech Scientific Ltd	Cat# 2-1045-250-IBA-250ML
10× Buffer R; Strep-Tactin ® Regeneration Buffer with HABA	Stratech Scientific Ltd	Cat# 2-1002-100-IBA-100ML
Bolt 4-12% SDS gel 17 well	Life Tech	Cat# NW04127BOX
1 kb DNA HyperLadder ™	Bioline	Cat# BIO33026
Trypan Blue	Sigma Aldrich Merck	Cat# T8154-100ML
Elite pre-stained protein ladder	Clinisciences	Cat# NB-45-00067-500
20× Bolt™ MES SDS Running Buffer	Life Tech	Cat# B000202
4X Bolt LDS Sample Buffer	Fisher Scientific	Cat# 13276499
DTT (Dithiothreitol)	Sigma Aldrich Merck	Cat# D0632-5G
cOmplete Protease Inhibitor Cocktail EDTA-free	Roche, purchased at Merck	Cat# 11873580001
Acetic acid	Fisher Scientific	Cat# 219992500
Chymotrypsin, Sequencing Grade	Promega	Cat# V1061
Triethylammonium bicaronate buffer (TEAB)	Merck	Cat# T7408
Biotin	Merck	Cat# B4639
PBS	Thermo Scientific	Cat# 10010001
TFA	Thermo Scientific	Cat# 293810250
Methanol	Thermo Scientific	Cat# 047192.K2
Acetonitrile	Thermo Scientific	Cat# 047138.K2
Formic Acid	Thermo Scientific	Cat# 147932500
NaCNBH_3_	Sigma Aldrich Merck	Cat# 156159-10G
CD3/CD28 Dynabeads	ThermoFischer	Cat# 40203D
Phorbol 12-Myristate 13-Acetate (PMA)	Promega	Cat# V1171

**Critical commercial assays**		

Prime star PCR Master Mix	Takara	Cat# R053A
Kinase-Ligase-Dpn1 enzyme mix	New England Biolabs	Cat# M0554S
HiPure Miniprep kit	ThermoFisher Scientific	Cat# K210002
MycoAlert™ kit	Lonza	Cat# LT07-318
LIVE/DEAD® Fixable Violet Dead Stain Kit	ThermoScientific	Cat# L34964
5kDa MWCO filters	Merck	Cat# MPE005025
Millex™-GP Filter Unit (0.22 μm Sterile)	Merck	Cat# SLGP033R
Millex™-HP Filter Unit (0.45 μm Sterile)	Merck	Cat# SLHP033R
Oasis PRiME HLB 3cc Vac Cartridge	Waters	Cat# WAT094226
Sep-Pak C18 3cc Vac RC Cartridge	Waters	Cat# WAT036945
Thermo Scientific Pierce C18 Spin Columns	ThermoFisher Scientific	Cat# 84850

**Experimental models: Cell lines**		

HEK293T	ATCC	Cat# CRL-1573
A549	ATCC	Cat# CCL-185
A549 MR1 KO clone 9 cells	ATCC and modified by Professor Andrew Sewell group	Laugel et al. 2016^8^
Jurkat TCR KO CD8αβ+ triple parameter reporter (TPR) cells transduced with MC.7.G5 TCR	Jurkats provided by Professor Peter Steinberger, Vienna, Austria. TCR transduction by Professor Andrew Sewell, Cardiff, Wales.	Dolton et al. 2025^9^

**Oligonucleotides**		

Strep-II forward oligo: 5′ CCGACAGATGGTCTCACCCCCAATTTGAAAAA CTCGAGAGCGGCTCCGGCGAG 3′	Eurofins Genomics	N/A
Strep-II reverse oligo: 5′ CTCTCGAGTTTTTCAAATTGGGGGTGAGACCA TCTGTCGGGGGTGGGCAGG 3′	Eurofins Genomics	N/A
K43A MR1 forward: 5′ GCCGAGCCCAGAGCCCCCTGG 3′	Eurofins Genomics	N/A
K43A MR1 reverse: 5′ GGCCTGTCTGGTCACGCTGTCG 3′	Eurofins Genomics	N/A
pELNS Forward: 5′ GAGTTTGGATCTTGGTTCATTC 3′	Eurofins Genomics	N/A
rat (r) CD2 Reverse: 5′ AACTTGCACCGCATATGCAT 3′	Eurofins Genomics	N/A

**Recombinant DNA**		

single chain (sc) codon-optimized gene for MR1 without its signal sequence (Uniprot.org, Nov. 2022) fused to β2M via a GGGGSGGGGSGGGGS linker	GeneArt	N/A
Codon-optimized A-F7 TCR was expressed from the pSF plasmid, with the TRA and TRB chains separated by a self-cleaving T2A, and a P2A between the TRB gene and rCD2 co-marker	Schmidlin et al. 2025^1^	N/A

**Software and algorithms**		

Skyline	MacLean et al. 2010^10^	https://skyline.ms/project/home/begin.view
FlowJo	Tree Star Inc.	https://www.flowjo.com/solutions/flowjo
Peaks v10.0	Bioinformatics Solutions	https://www.bioinfor.com/
MSconvert	ProteoWizard	https://proteowizard.sourceforge.io/download.html
Rawrrr	Kockmann et al. 2021^11^	https://bioconductor.org/packages/3.21/bioc/html/rawrr.html
LigandScanner	This paper	https://doi.org/10.5281/zenodo.17689426
CEU Mass Mediator	Garcia et al. 2016^12^	https://ceumass.eps.uspceu.es/
Biorender	Biorender	https://www.biorender.com/
NovoExpress	Agilent	https://www.agilent.com/en/product/research-flow-cytometry/flow-cytometry-software/novocyte-novoexpress-software-1320805

**Other**		

BD FACSCanto II	BD Biosciences	https://www.bdbiosciences.com/en-se/products/instruments/flow-cytometers/clinical-cell-analyzers/facscanto
ACEA NovoCyte 3005 with NovoSampler pro	ACEA, Agilent	Legacy instrument
Strep-Tactin XT 4flow column	IBA Lifesciences	https://www.iba-lifesciences.com/strep-tag
ӒKTA Pure protein purification system	Cytiva	https://www.cytivalifesciences.com/en/us/shop/chromatography/chromatography-systems/akta-pure-p-05844?srsltid=AfmBOoqR6S7Wc8wa8GLqsh6Zrcjraya-ZnXBvSWJekO4cZ83TL6Pryrw
Q Exactive HF-X mass spectrometer	Thermo Scientific	Legacy instrument
Ultimate 3000 RSLCnano System	Thermo Scientific	Cat# ULTIM3000RSLCNANO
Acclaim PepMap 100 C18 5 μM 0.1×20 mm column	Thermo Scientific	Cat# 164946
75μm×50cm PepMap RSLC C18 EasySpray	Thermo Scientific	Cat# ES75500PN
StageTips (C18)	Rappsilber *et al.*, 2007^13^	N/A

## Materials and equipment

### Solutions to be prepared in advance

• **10 mM triethylammonium bicarbonate buffer (TEAB):** Mix 9 mL LC-MS grade water with 1 mL 1 M TEAB. Store at 4 °C.

***Note:*** Diluted triethylammonium bicarbonate (TEAB) is volatile and may undergo pH drift over time. In our experience, diluted TEAB remains stable for up to 1–2 weeks when stored at 4 °C in tightly sealed containers.

• **2 mM detachment medium:** Add 2 mL 0.5 M EDTA using a 0.22 μm syringe filter into a bottle of 498 mL D-PBS. Store at 4 °C for maximum 1 month. Pre-warm at 37 °C before use.
• **Fixing buffer:** Add 54 mL formaldehyde to 446 mL D-PBS using a 0.22 μm filter syringe (4% paraformaldehyde). Store at 4 °C.
• **Freezing buffer:** Add 5 mL DMSO to 45 mL Fetal Bovine Serum (FBS) using a 0.22 μm filter syringe (10% DMSO). Store at 4 °C.

**Table:** 50× TAE buffer

Reagent	Final concentration	Amount
Trizma® base	50×	242 g
1 M acetic acid	57.1 mM	57.1 mL
0.5 M EDTA	50 mM	100 mL
MilliQ Ultrapure water	N/A	Up to final vol
**Total**	**N/A**	**1 L**

[Adjust pH to 8].

**Table:** R10 media

Reagent	Final concentration	Amount
RPMI-1640	N/A	435 mL
Penicillin, Streptomycin	100 U/mL, 100 μg/mL	10 mL
L-Glutamine	2 mM	5 mL
FBS	10%	50 mL
**Total**	**N/A**	**500 mL**

[Filter through 0.22 μm filter syringe. Store at 4 °C for maximum 1 month. Pre-warm at 37 °C before use.].

**Table:** D10 media

Reagent	Final concentration	Amount
Dulbecco’s Modified Eagle Medium (DMEM)	N/A	435 mL
Penicillin, Streptomycin	100 U/mL, 100 μg/mL	10 mL
L-Glutamine	2 mM	5 mL
FBS	10%	50 mL
**Total**	**N/A**	**500 mL**

[Filter through 0.22 μm filter syringe. Store at 4 °C for maximum 1 month. Pre-warm at 37 °C before use.].

### Solutions to be prepared on the day of the experiment

**Table:** Lysis buffer

Reagent	Final concentration	Amount
5 M NaCl	150 mM	1.5 mL
Tris base	50 mM	0.303 g
10% NP-40	0.5%	2.5 mL of 10%
MilliQ Ultrapure water	N/A	Up to 50 mL
cOmplete Protease Inhibitor	1×	1 tablet
**Total**	**N/A**	**50 mL**

[Adjust pH to 7.4 using 1 M HCl if needed].

**Table:** 1% agarose gel

Reagent	Final concentration	Amount
UltraPure™ Agarose	1%	1 g
1× TAE buffer	1×	100 mL
**Total**	**N/A**	**100 mL**

• **1× running buffer for SDS-page gel:** Use 50 mL of 20× Bolt™ MES SDS running buffer diluted to 1 L with MilliQ Ultrapure water.
• **SDS reducing sample buffer:** Make as much as required to run your samples. 2 μL 4X Bolt LDS sample buffer + 2 μL Dithiothreitol (DTT) per sample.
• **NaCNBH**_**3**_ **stock solution (0.5 M in 10 mM TEAB):** Weigh in 15.71 mg NaCNBH_3_ into a 15 mL Eppendorf tube and resuspend in 500 μL 10 mM TEAB by vortexing.

**CRITICAL:** Sodium cyanoborohydride (NaCNBH_3_) is a toxic and moisture-sensitive reducing agent. It can release hydrogen cyanide (HCN) upon contact with acids. Always handle NaCNBH_3_ inside a certified fume hood, wearing appropriate PPE (lab coat, gloves, safety glasses), avoid inhalation of dust, and keep the reagent bottle open only for the shortest possible time. Dispose of all NaCNBH_3_-containing waste according to institutional hazardous-chemical guidelines.

***Note:*** To minimize handling time and reduce exposure to dry NaCNBH_3_, we routinely prepare the stock solution by placing an empty 1.5 mL microcentrifuge tube on a balance in the hood, taring the weight, and then adding a small amount of reagent directly into the tube with a dedicated spatula. The weight of NaCNBH_3_ is recorded, and the appropriate volume of TEAB is added to reach the desired final concentration. This approach reduces manipulation of the stock container and shortens exposure time. All weighing should be performed in a fume hood.

## Step-by-step method details

### Design, cloning, and site-directed mutagenesis of scMR1 constructs

**Timing: 3 weeks**

This section describes the design of the single chain (sc)MR1 construct, a strep-II-tagged, single chain MR1/β2M fusion protein, to be later used for transduction into A549 cells and the process of site-directed mutagenesis required to mutate the MR1 lysine 43 to an alanine (K43A). This mutation serves as a negative control, as it prevents ligands from forming a covalent Schiff base with MR1 in the binding groove.

1. Design the single chain (sc)MR1 construct (e.g., using GeneArt) to include the following components: a codon-optimized MR1 gene lacking its native signal sequence (using resources from UniProt.org) fused to β2M with a GGGGSGGGGSGGGGS linker.
2. Perform site directed mutagenesis (SDM) to add a Strep-II tag to the scβ2M/MR1 construct.
  a. Order the following oligonucleotides:
    i. Strep-II forward oligo
      (5′ CCGACAGATGGTCTCACCCCCAATTTGAAAAACTCGAGAGCGGCTCCGGCGAG 3′).
    ii. Strep-II reverse oligo
      (5′ CTCTCGAGTTTTTCAAATTGGGGGTGAGACCATCTGTCGGGGGTGGGCAGG 3′).
  b. Perform PCR using the Prime star PCR Master mix with cycling conditions according to the manufacturer’s instructions, but including a 1 min per kb elongation step.
  c. Run the PCR products on a 1% agarose gel with a 1 kb DNA HyperLadder™ at 90 V for 50 minutes.
  d. Treat correct PCR products with a kinase-ligase-Dpn1 enzyme mix for 30 minutes at 20-25 °C.
  e. Transform samples into XL10-Gold Ultracompetent cells using heat shock.
  f. Plate these cells onto an LB-Agar plate containing 50 μg/mL Carbenicillin antibiotic.
  g. Identify antibiotic resistance colonies and amplify their plasmid DNA using a HiPure Miniprep kit as per manufacturer’s instructions.
3. Submit the DNA samples for sequencing (e.g., Eurofins Genomics) to confirm the SDM has worked using the following primers:
  a. Forward primer: 5′ GAGTTTGGATCTTGGTTCATTC 3′
  b. Rat(r)CD2 reverse primer: 5′ AACTTGCACCGCATATGCAT 3’.
4. Repeat from Step 2 to make the scMR1 K43A substitution.
  a. Order and use the following oligonucleotides:
    i. K43A MR1 forward (5′ GCCGAGCCCAGAGCCCCCTGG 3′)
    ii. K43A MR1 reverse (5′ GGCCTGTCTGGTCACGCTGTCG 3′).
5. Submit the scMR1-K43A DNA sample for sequencing (e.g., Eurofins Genomics) using the same primers as used in Step 3.

### Preparation of MR1-expressing cell lines

**Timing: 3–4 weeks**

This section describes the generation of MR1-lentivirus using HEK293T cells and viral transfection of A549 MR1 KO cells. These cell lines overexpressing the scMR1-WT and the scMR1-K43A mutation allow the eventual purification of MR1-bound ligands via Strep-Tactin® enrichment and eventual identification of candidate ligands.

6. Plate HEK293T cells for transfection (24 hours prior)
  a. Detach HEK293T cells as described in culture preparation.
  b. Count the cells using a haemocytometer in a 1:1 ratio of trypan blue.
  c. Plate 1.25 × 10^6^ cells per well of a 6-well plate in a total of 3 mL D10 media per well.
7. Make the scMR1 strep-II plasmid mix for HEK293T transfection.
  a. Mix the following amounts of DNA plasmids in an Eppendorf tube

**Table:** 

Reagent	Concentration per 6-well plate (1× well)
scMR1-rCD2-pELNS plasmid	1.52 μg
pMD2.G plasmid	0.72 μg
pMDLg/pRRE plasmid	1.83 μg
pRSV-REV	1.83 μg
Polyethylenimine	18 μg
Opti-MEM™	Up to 300 μL
  b. Incubate at 20-25 °C for 15 minutes.
8. Transfection of HEK293T cells
  a. Add the 300 μL plasmid mixture from Step 7 to the 6-well of pre-plated HEK293T cells in a gentle drop-wise manner.
  b. Incubate the transfected HEK293T cells for 24 hours at 37 °C in a 5% CO_2_ environment.
  c. Remove and replace the media with 3 mL fresh D10 media.
  d. Collect the supernatant 48 and 72 hours post transfection
    i. Combine the 2 time-point supernatants
    ii. Filter through a 0.45 μm polyethersulfone membrane filter.

**Pause point:** The filtered lentiviral supernatant can either be used immediately to transfect the cell line of choice or can be preserved at −80 °C for long term storage.

***Note:*** If lentiviral supernatant is frozen, it must be thawed on ice on the day of use. Lentivirus can only be thawed once per use and cannot be refrozen, so freeze in aliquots of 1-2 mL for storage.

**CRITICAL:** The post-transfection cell supernatant contains the lentivirus for MR1 transduction, so it is important to harvest all supernatants at 48 and 72 h post HEK293T transfection. Filtering this media using 0.45 μm pore size filters removes any contaminating HEK293T cells.

9. Plate A549 MR1 KO cells for transfection (24hrs prior)
  a. Detach A549 MR1 KO cells as described in culture preparation.
  b. Count the cells using a haemocytometer in a 1:1 ratio of trypan blue.
  c. Plate 0.1–0.2 × 10^6^ cells per 1× well in a 24-well plate in a total of 2 mL R10 media per well.

***Note:*** The A549 MR1 KO cells are cultured in this protocol for two reasons; to be transduced with scMR1-WT or scMR1-K43A lentivirus and to serve as a negative control in validation assays later. Maintain these cells in culture throughout the experimental pipeline.

10. Perform Spinfection of A549 MR1 KO cells for lentiviral transduction
  a. Immediately prior to transduction, remove the R10 medium.
  b. Add 0.5–1 mL fresh R10 media with a 1:1 volume ratio of either fresh or thawed lentiviral supernatant.
  c. Add 5 μg per mL of polybrene to the cells.
  d. Centrifuge the cells with their lentiviral supernatant (and polybrene) at 400 *× g* for 2 hours for spinfection.
  e. Without disturbing the spinfected cells (with lentivirus and polybrene), incubate at 37 °C in a 5% CO_2_ environment for 14-16 hours.
  f. Aspirate the medium and replace with fresh R10.
  g. Maintain these transduced A549 MR1 KO cells (to allow expression of scMR1-WT or scMR1-K43A) in culture for 7 days post-transduction.
  h. Assess MR1 expression by surface antibody staining (see Step 31).

***Note:*** Lentiviral production and transduction of cell lines use genetically modified (GM) plasmid DNA. Store all GM materials in designated areas according to institutional guidelines. Treat waste with 2500 ppm chlorine disinfectant for at least 4 h before autoclaving. Send autoclaved waste for incineration. Keep treated materials a Category II safety cabinet for the full decontamination period.

11. Enrich transduced MR1-expressing cell lines.
  a. Detach A549 MR1 KO + scMR1-WT and A549 MR1 KO + scMR1-K43A cells.
  b. Count the cells using a haemocytometer in a 1:1 ratio of trypan blue.
  c. Transfer 1 × 10^6^ of each cell line into a FACS tube.
  d. Wash the cells:
    i. Add 4 mL D-PBS.
    ii. Centrifuge at 400 *× g* for 5 minutes.
    iii. Remove the supernatant.
  e. Stain the cells with LIVE/DEAD® Fixable Violet Dead Stain Kit:
    i. Add 2 μL of a 1:40 dilution in D-PBS to each well
    ii. Vortex.
    iii. Incubate cells for 5 minutes at 20-25 °C in the dark.
  f. Add 1 μL MR1-PE antibody (clone 26.5, 2 μg/mL, BioLegend) per FACS tube, vortex the tubes and incubate for 20 minutes on ice in the dark.
  g. Wash once more as described in Step 11d.
  h. Sort the live MR1-positive cells using a BD FACS Aria III, using a final volume of 100 μL D-PBS per sample
  i. Culture the sorted cells for downstream applications or preserve as frozen stocks.

***Note:*** Use this cell sorting step to isolate MR1-expressing cells confirmed by surface staining. Prior to sorting, expand the transduced cells from the initial 24-well transfection into T75 flasks to obtain adequate numbers.

**Pause point:** After generating MR1-expressing cells, they can be cryopreserved for long term storage. Cells can be stored for years in the vapor phase of liquid nitrogen.

***Optional:*** Cryopreservation of MR1-expressing cells: (i) Detach A549 MR1 KO + scMR1-WT and A549 MR1 KO + scMR1-K43A cells and count the cells using a haemocytometer in a 1:1 ratio of trypan blue. (ii) Resuspend 1 × 10^6^ cells per 1 mL of freezing buffer and freeze using Mr Frosty or CoolCell freezing pots/containers according to manufacturer’s instructions to achieve an approximate cooling rate of −1 °C/min. (iii) To resume cell culture, thaw rapidly at 37 °C and reseed into 12 mL prewarmed R10 medium in a T75 culture flask.

### Cell growth, lysis, and MR1 enrichment

**Timing: 2–3 weeks for growth and 1 day for lysis and MR1 enrichment**

**Figure 2:**
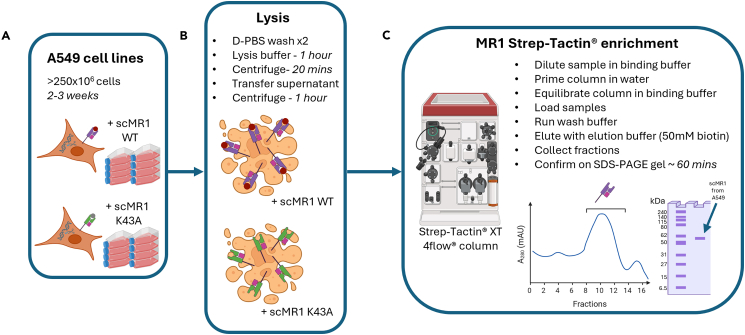
Culture for growth, lysis and MR1-enrichment from A549 cells (A) A549 MR1 KO cells expressing either scMR1-WT or scMR1-K43A are cultured over 2-3 weeks until high numbers of cells (>250 × 10^6^) are reached. (B) highlights the key steps of the lysis process in order to isolate the scMR1 molecules. (C) describes the basics of Strep-Tactin® enrichment, using a FPLC machine with Strep-Tactin® XT 4flow® columns. Results are expected as a clear peak on a chromatography trace. Fractions containing the peak will be loaded and run on an SDS-PAGE gel for size confirmation of scMR1 molecules.

This section describes expansion of A549 cells to generate sufficient biomass for bulk lysis to isolate and purify scMR1-ligand complexes using Strep-Tactin®. Due to low MR1 expression levels even in overexpression systems, large quantities of cells are needed to obtain sufficient MR1 for successful ligand detection, best achieved by bulking up approximately 2.5 × 10^8^ cells for each desired cell line over the course of 2-3 weeks (Figure 2).

12. Prolonged culture of scMR1-expressing A549 cell lines
  a. Grow A549 MR1 KO + scMR1-WT and A549 MR1 KO + scMR1-K43A cells in multiple T175 culture flasks as described in culture preparation.
  b. Count the total number cells at regular intervals to predict when you will have ∼2.5 × 10^8^ cells for lysis.
13. Lysis of A549 MR1 KO + scMR1-WT and A549 MR1 KO + scMR1-K43A cell lines
  a. Detach A549 cells as described in culture preparation and count the cells using a haemocytometer in a 1:1 ratio of trypan blue.
    **CRITICAL:** For cell lysis, a very large quantity of the A549 cells is required so this should be considered when culturing new MR1-expressing cell lines. Bulking up these cell lines may take several weeks.
    ***Note:*** The cells to be lysed in this step include: A549 MR1 KO + scMR1-WT and A549 MR1 KO + scMR1-K43A, which collectively provide all conditions required for comparative LC-MS analysis.
  b. Harvest the cells into a centrifuge tube:
    i. Centrifuge at 400 *× g.*
    ii. Remove the supernatant.
    iii. Resuspend in R10 medium.
    iv. Count cells using a haemocytometer and a 1:1 ratio of trypan blue.
  c. Transfer 250 × 10^6^ of each desired cell line to a new 50 mL centrifuge tube with 50 mL cold D-PBS.
  d. Centrifuge at 400 *× g* for 5 minutes.
  e. Repeat this 50 mL D-PBS wash once more (total 2x 50 mL washes).
  f. Resuspend cells in 10-15 mL lysis buffer (see Materials).
  g. Add 0.03 mg Strep-Tactin®.
  h. Incubate the lysing cells on ice for 1 hour then centrifuge at 400 *× g* for 20 minutes at 4 °C.
    ***Note:*** The 20-min centrifugation allows for all lysed cellular debris to pellet. If the supernatant does not look clear, the centrifugation can be repeated.
  i. Carefully remove all the supernatant without disturbing the debris pellet to a clean centrifuge tube.
  j. Centrifuge this supernatant at 12,000 *× g* for 1 hour at 4 °C.
    **Pause point:** The supernatant can be stored at 4 °C for 14-16 h or −20 °C for up to 1 week.
14. Strep-Tactin® enrichment of MR1 from A549 MR1 KO + scMR1-WT and A549 MR1 KO + scMR1-K43A cells
  a. Dilute 1:10 with MilliQ Ultrapure water, to make a 1× working concentration of the 10× Strep-Tactin® wash buffer.
  b. Dilute the supernatant 1:4 with Strep-Tactin® wash buffer and 0.45 μm filter into a clean Duran bottle.
  c. Use a fresh (new or fully regenerated) 1 mL Strep-Tactin® XT 4flow® column for each A549 cell line.
  d. Couple the columns to an ӒKTA Pure protein purification system using a top wet connection.
  e. Prime each column with a minimum of 4 column volumes (CV) of MilliQ Ultrapure water at a 3 mL per min flowrate.
  f. Equilibrate each column with a minimum of 4 CV Strep-Tactin® wash buffer at a flow rate of 5 mL per min.
  g. Load the filtered cell supernatant(s) from step 13 at 1 mL per min.
  h. Wash with 8 CV Strep-Tactin® wash buffer at 5 mL per min.
  i. Elute the bound MR1 with 6 CV elution buffer (containing 50 mM biotin) with a flow rate of 5 mL per min, collecting 1 mL fractions.
  j. Run 3 CV of CV MilliQ Ultrapure water, regeneration buffer, MilliQ Ultrapure water and finally 20% ethanol to clean the columns.
  k. Store the columns at 4 °C.

***Note:*** The UV FPLC trace typically peaks around fractions 10-14, where MR1 elutes, although this may vary between different FPLC setups.

**CRITICAL:** The Strep-Tactin® XT system is required for efficient enrichment of scMR1. Substitution with alternative affinity resins has not been validated and may result in reduced recovery.

15. Run an SDS-PAGE gel with the 1 mL fractions to test for MR1-ligand complexes at the expected molecular weight.
  a. Set aside 20 μL of each fraction.
  b. Add 4 μL SDS reducing sample buffer to each sample.
  c. Incubate samples at 70 °C for 10 minutes.
  d. Centrifuge the samples at 16,000 *× g* for 30 seconds.
  e. Load the samples onto BOLT 4-12% Bis-Tris gels.
  f. Load 5 μL Elite pre-stained protein ladder in a separate well.
  g. Run the gel at 165 V for 45 minutes in 1× BOLT MES SDS-PAGE running buffer.
  h. Stain your finished gel with 20 mL quick Coomassie stain until bands appear.
  i. Destain the gel with water.

***Note:*** Larger bands are often observed in MR1 enrichment fractions. The scMR1-β2M complex should appear around 60 kDa.

### Cross-link MR1-ligand complexes by reductive amination

**Timing: ∼4–6 h**

This section describes the stabilization of affinity-enriched MR1–ligand complexes by reductive amination, which converts the reversible Schiff base between MR1 and its ligand into a stable secondary amine using sodium cyanoborohydride (NaCNBH_3_). Prior to reduction, samples are exchanged into TEAB, and after cross-linking, excess reagent is removed by buffer exchange to prepare the sample for chymotryptic digestion and downstream LC–MS/MS analysis.

16. Exchange enriched MR1 samples to remove excess biotin from the elution buffer.
  a. Transfer the enriched MR1 sample onto a 5 kDa MWCO filter and perform buffer exchange into 10 mM TEAB according to the manufacturer’s instruction.
  b. Discard the filtrate.
  c. Add fresh 10 mM TEAB to the retentate.
  d. Repeat 2–3 buffer-exchange cycles to effectively remove free biotin carried over from the Strep-Tactin® elution.
  e. Recover the MR1 concentrate:
    i. Invert the device into a fresh collection tube.
    ii. Spin at ∼1,000 × g for 3 minutes

**CRITICAL:** Ensure the filter does not run dry during centrifugation, as this can lead to irreversible sample loss. To prevent this, centrifuge the filter device in multiple shorter pulses (e.g. 3–5 min each) rather than a single prolonged centrifugation step. Visually monitor the retentate volume after each spin and either add fresh buffer or proceed to the recovery step, before the membrane becomes exposed.

***Note:*** The purpose of this buffer-exchange step is to remove biotin used during MR1 elution, as residual biotin interferes with downstream LC–MS/MS analysis. We typically use the 5 kDa Microcon® Biomax® PES filter for this step but other 5 kDa or 10 kDa molecular-weight cutoff devices are suitable depending on sample volume and laboratory workflow preferences.

17. Add sodium cyanoborohydride (NaCNBH_3_) to initiate reductive amination.
  a. Prepare a fresh NaCNBH_3_ stock solution in 10 mM TEAB immediately before use.
  b. Add NaCNBH_3_ stock solution to the MR1 sample to reach a final concentration of 20–50 mM.
  c. Incubate the reaction for 2 hours at 20-25 °C with gentle agitation (approximately 50 rpm)

**CRITICAL:** This workflow relies on using NaCNBH_3_ for reductive amination to stabilize labile Schiff base MR1–ligand adducts. NaCNBH_3_ is a mild, imine-selective reducing agent that performs well under the described buffer conditions. Substituting with other reducing agents may lead to lower conversion efficiency or increased nonspecific reduction.

18. Exchange cross-linked MR1 samples into PBS for chymotryptic digestion.
  a. Transfer the cross-linked MR1 sample from the reductive-amination reaction onto a 5 kDa MWCO filter.
  b. Perform buffer exchange into 1 × PBS (pH 7.4) according to the manufacturer’s instruction.
  c. Repeat the buffer-exchange cycle 2-3 times to efficiently remove residual NaCNBH_3_.
  d. Recover the MR1 concentrate:
    i. Invert the device into a fresh collection tube.
    ii. Spin at ∼1,000 × g for 3 minutes.
  e. Adjust final volume with 1×PBS to approximately 200-300 μL

***Note:*** This buffer-exchange step serves to remove NaCNBH_3_ and TEAB and place the cross-linked MR1-ligand complexes into PBS, which is compatible with subsequent chymotrypsin digestion.

**CRITICAL:** Ensure that buffer exchange is performed with at least 2-3 PBS washes to minimize carryover of NaCNBH_3_, which can result in additional side reactions and interfere with downstream enzymatic digestion. As above, ensure the filter never runs dry, as this can lead to irreversible sample loss.

### Digest MR1 complexes with chymotrypsin and desalt peptides

**Timing: ∼12–20 h (14–16 h digestion followed by 2–3 h desalting)**

This section describes the proteolytic digestion of cross-linked MR1–ligand complexes using chymotrypsin, which reliably generates the MR1-derived peptide DSVTRQKEPRAPW containing the ligand-modified lysine (K43) required for Δ-mass analysis. Following digestion, which is performed without any reduction or alkylation to preserve the cross-linked ligand, peptides are desalted to remove residual salts prior to LC–MS/MS analysis.

19. Prepare chymotrypsin according to the manufacturer’s instructions (e.g., Promega sequencing-grade chymotrypsin).

***Note:*** After reconstitution, chymotrypsin can be aliquoted and stored at −80 °C. Use freshly reconstituted enzyme or a freshly thawed aliquot for each experiment. Avoid repeated freeze-thaw cycles to prevent loss of activity.

***Note:*** Sequencing-grade chymotrypsin from other commercial suppliers may be used, provided comparable activity and specificity are ensured.

20. Add chymotrypsin to the sample at the recommended enzyme-to-protein ratio.

***Note:*** Most manufacturers recommend enzyme-to-protein ratios of 1:200 to 1:20 (w/w). In this workflow, digestion efficiency is considered optimal when reproducible peptide profiles and robust detection of MR1-derived peptides are achieved, rather than complete proteolysis. If total protein amount is not known, use a fixed amount of chymotrypsin based on the starting material. In our workflow, samples derived from ∼2.5 × 10^8^ cells typically require 200 ng of chymotrypsin (adjust based on empirical digestion efficiency).

21. Incubate for 16 hours at 25 °C with gentle shaking (e.g., 50 rpm; follow manufacturer instructions).
22. Acidify the reaction with TFA to a final pH range of 2–2.5 to stop digestion.

***Note:*** When working with sample volumes of 200-300 μL in PBS, add TFA in 1 μL increments and mix thoroughly after each addition. Monitor pH by spotting 1 μL of the reaction mix onto universal indicator paper. Repeat until the desired pH range is reached. In our experience, a total of ∼3–5 μL TFA is typically required.

**CRITICAL:** TFA is toxic and volatile. Perform all handling in a certified fume hood and wear appropriate personal protective equipment.

23. Desalt peptides using C18 reversed-phase material (e.g., Waters Sep-Pak, or OASIS PRiME HLB)
  a. Clarify the acidified digest by brief centrifugation (e.g., 10 min at 20,000 *× g*) to remove insoluble debris.
  b. Condition and equilibrate the C18 material according to the manufacturer’s instructions.
    ***Note:*** Typical steps include activation with 1 column volume (CV) MeOH or acetonitrile (ACN) followed by equilibration with 2 CV of 0.1% FA / 2% ACN in water.
  c. Load the acidified peptide solution onto the equilibrated C18 material. Allow full binding by gravity flow, low vacuum, or gentle centrifugation as appropriate.
  d. Wash the C18 material with 2 CV of 0.1% FA/2% ACN in water.
  e. Elute peptides with 1 CV of 0.1% FA/80% acetonitrile into a clean low-binding tube.
  f. Dry eluates *in vacuo.*
    **Pause point:** In dried form, desalted peptide samples can be stored at −80 °C for at least 2 months. Avoid repeated freeze**-**thaw cycles; aliquot samples before drying if multiple LC–MS/MS analyses are anticipated.
  g. Reconstitute peptides in LC loading buffer (typically 0.1% formic acid) for LC–MS/MS analysis.
    ***Note:*** Sep-Pak cartridges, OASIS HLB, StageTips, and MicroSpin C18 columns (e.g. Pierce C18 Spin Columns) are all compatible with this workflow. Choose the device that matches your sample volume and available equipment. Ensure that the final reconstitution volume is compatible with your LC injection volumes and with the expected MR1 enrichment yield.
    ***Note:*** Ligand modification can alter the retention behavior of the MR1-derived peptide DSVTRQKEPRAPW during C18 desalting. Highly polar or multiply acidic modifications (e.g., phosphate- or sulfonate-containing ligands) may reduce retention, whereas strongly hydrophobic modifications may increase retention. If recovery of ligand-modified MR1 peptides is low, (i) avoid overly stringent wash conditions and keep organic solvent content low during loading and washing, and (ii) consider slightly stronger elution conditions to ensure recovery of more hydrophobic species (e.g. by increasing the organic content of the elution buffer up to 100% or by eluting in higher volumes).

### Acquire LC-MS/MS data

**Timing: 60–70 min per injection**

This step acquires high-resolution MS/MS spectra of chymotryptic MR1 peptides that carry covalently stabilized small-molecule ligands. Any high-resolution, state-of-the-art LC-MS system can be used. The settings below reflect those used in Schmidlin et al.^1^ performed on a Q Exactive HF-X mass spectrometer coupled to an Ultimate 3000 RSLCnano System (both Thermo Scientific), representing typical Orbitrap settings.

24. Separate chymotryptic peptides by reverse-phase LC.

***Note:*** For peptide separation we favor the use of a trap-elute setup using the Acclaim PepMap 100 C18 5 μM 0.1 × 20 mm precolumn for trapping and the PepMap RSLC C18 EasySpray column 75 μm × 50 cm for separation. We prefer mobile phases containing 1% (v/v) DMSO in mobile phase A and B to enhance ionization efficiency and using 0.1% (v/v) FA as an acidic modifier. Ensure samples are trapped in 2% ACN or less. A linear gradient from 2% to 30% ACN over 60–70 min efficiently resolves MR1-derived peptides. Slightly steeper gradients may reduce run time without compromising separation.

25. Analyze eluting peptides by MS.

**CRITICAL:** Acquire spectra in data-dependent acquisition (DDA) mode to minimize chimeric MS/MS spectra. The current Δ-mass analysis pipeline is optimized for DDA data and does not support the deconvolution of chimeric spectra typically generated in data-independent acquisition (DIA) workflows. DDA acquisition reliably captures ligand-modified MR1 peptides and has been used successfully for both novel and known MR1 ligands (Schmidlin et al.^1^). Parallel reaction monitoring (PRM) is compatible with this workflow and provides an effective alternative for targeted analyses of known MR1 ligands.

***Note:*** For mass spectrometric analysis of peptides we recommend the following settings on the Q Exactive HF-X: Use a spray voltage of 2.0 kV (depending on the emitter setup used), with an ion transfer capillary temperature of 305 °C, full MS resolution set to 60,000 at *m/z* 200 and a scan range of 320–1600 *m/z*. Acquire fragment ion spectra for selected precursors using HCD fragmentation for the range 200 to 2000 *m/z* at a resolution of 60,000. Use normalized collision energy (nCE) of 25, which has been shown to provide robust fragmentation of the MR1-derived peptide DSVTRQKEPRAPW. Set DDA selection criteria to top 12 precursors with a minimum intensity of 850 and charge states 2-4, using a 1.3 amu isolation window, and a dynamic exclusion of 30s.

### Process LC-MS/MS data and extract Δ-mass events

**Timing: ∼2–3 days**

This section describes the processing of raw LC–MS/MS data to identify candidate MR1 ligands by converting proprietary MS files into open formats and screening them for MR1-K43-associated Δ-mass modifications using LigandScanner, a custom *R* script provided with this protocol. Resulting ligand masses are annotated against multiple compound databases via CEU Mass Mediator^12^ enabling systematic shortlisting of plausible ligand candidates for subsequent biochemical validation.

26. Convert .raw MS files into .mgf files using MSConvert.
  a. Add your file into MSConvert
  b. Set Output format to .*mgf* and enable *Write index*, *Use zlib compression*, and *TPP compatibility*.
  c. Add filters for Peak Picking = *Vendor*; Threshold Peak Filter = *100 most intense* ions.
  d. (Optional) Apply a *Scan time (seconds)* filter corresponding to the expected retention time window of the ligand-modified DSVTRQKEPRAPW peptide:

***Note:*** Set this to range from 2 min before to 10 min after the retention time of the free DSVTRQKEPRAPW (as determined in your database-search results). This limits processing to the chromatographic region where ligand-modified peptide variants are expected to elute.

***Note:*** Ligand cross-linking slightly increases hydrophobicity; therefore, modified MR1 peptides often elute later than the unmodified peptide. Extending the post-RT window captures these shifted species while keeping file size and analysis time low.

**CRITICAL:** When .mgf export is performed without centroiding, each MS/MS spectrum is written as a dense ion list that mirrors the instrument’s native profile resolution. This inflates file size, produces redundant fragment peaks, and reduces identification accuracy. Enabling Peak Picking = *Vendor* converts spectra to centroided fragment ion lists suitable for Δ-mass analysis. Using the Top 100 most intense filter further reduces file size and improves computational performance while preserving all relevant signals.

27. Download and prepare LigandScanner for Δ-mass analysis.
  a. Download LigandScanner.R from Zenodo (https://doi.org/10.5281/zenodo.17689426). Save the script and your .mgf file in a dedicated analysis directory (e.g., /analysis/delta_mass/)
  b. Open LigandScanner in RStudio and replace filename in line 23 to point to the .mgf file generated in Step 26
28. Run LigandScanner and locate the output file named *LigandScannerAbsolute.csv* in your analysis folder.

***Note:*** Example .raw files from our study can be downloaded from peptide atlas using dataset identifier PASS05867. The script extracts and counts reporter ions, indicative of K43-associated Δ-mass shifts for each MS/MS spectrum in the analysis. For each spectrum it reports: presence/absence of individual reporter ions, total reporter ion count (labelled #Fragments), Δ-mass value, and inferred molecular mass of the free ligand (labelled “Delta_Mass_Oxygen”).

**CRITICAL:** The script comes with generic filter settings. It applies a minimum Δ-mass threshold of 40 Da, as MR1 rarely binds ligands below this size (Schiff base stabilized glyoxal yields a 42 Da shift). Adapt the parameter Min_mass_shift in line 18 if you wish to use a different threshold. Additionally, we recommend to prioritize K43-associated Δ-mass events supported by the highest number of reporter ions. The optimal reporter ion cutoff depends on overall data quality, which is influenced by MR1 enrichment efficiency, ligand occupancy, and LC–MS settings. Our script works with a generic cutoff value of 8. Adapt the parameter Min_frag_number on line 19 if you wish to use a different threshold.

**Figure 3:**
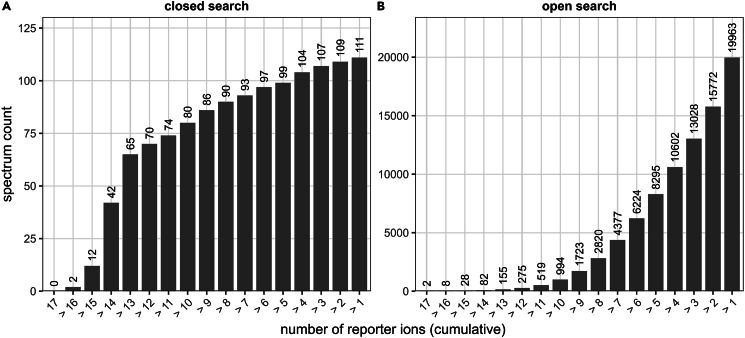
Cumulative fragment ion coverage in closed and open search workflows (A) Cumulative number of spectra identified in the closed search, plotted as spectrum count across decreasing fragment ion thresholds. (B) Cumulative number of spectra identified in the open search, plotted using the same fragment ion thresholds. Bars represent cumulative reporter ion counts (≥ n fragments), and values above each bar indicate the exact cumulative spectrum count. Results are taken from one LC-MS/MS run of cross-linked scMR1 enriched from A549 cells. The figure shows trends described for open search (plateau/only slow increase when stepwise relaxing thresholds below 13 reporter ions) and closed search (exponential growth of spectral count with lower stringency).

***Optional:*** To improve confidence in Δ-mass assignment, apply a reporter ion threshold derived from the reporter ion distribution of the unmodified MR1 peptide (DSVTRQKEPRAPW, Δ-mass ≈ 0). In the original workflow (Schmidlin et al.^1^), a closed and an open search were compared to determine a data-driven cutoff. In the closed search (Δ-mass fixed at 0 ± instrument error), lowering the minimum number of required reporter ions eventually leads to a lower rise or even a plateau in identifications, reflecting the high spectral quality of the abundant unmodified peptide. In contrast, the open search (no Δ-mass restriction) shows an exponential increase in identifications as stringency decreases. The reporter ion threshold located at the transition between these two regimes - where the closed search plateaus and the open search begins to rise steeply - provides a practical cutoff for excluding low-confidence Δ-mass assignments. This works best when multiple replicates are merged (Figure 3).

**Figure 4:**
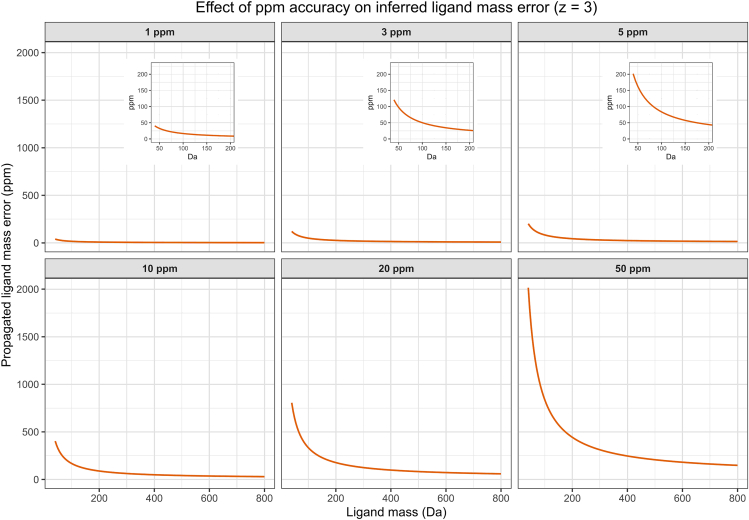
Propagation of precursor mass error to inferred ligand mass estimates Propagated ligand mass error as a function of ligand size (40–800 Da) across six mass-accuracy settings (1, 3, 5, 10, 20, and 50 ppm) for the ligand-bound MR1 peptide DSVTRQKEPRAPW (z = 3 precursor ions). Each panel shows the inferred ligand-mass deviation resulting from the corresponding MS1 precursor mass tolerance, illustrating that even small precursor errors disproportionately affect ligand-mass estimation for low-mass adducts. Insets in the 1, 3, and 5 ppm panels provide a zoom into the low-mass region (40–200 Da), demonstrating that substantial propagated error persists even at high mass accuracy (≤3 ppm). Overall, the figure highlights the need for wider ligand-mass tolerance settings during candidate screening and provides a reference for selecting appropriate CEU Mass Mediator parameters and downstream data-filtering thresholds.

**Figure 5:**
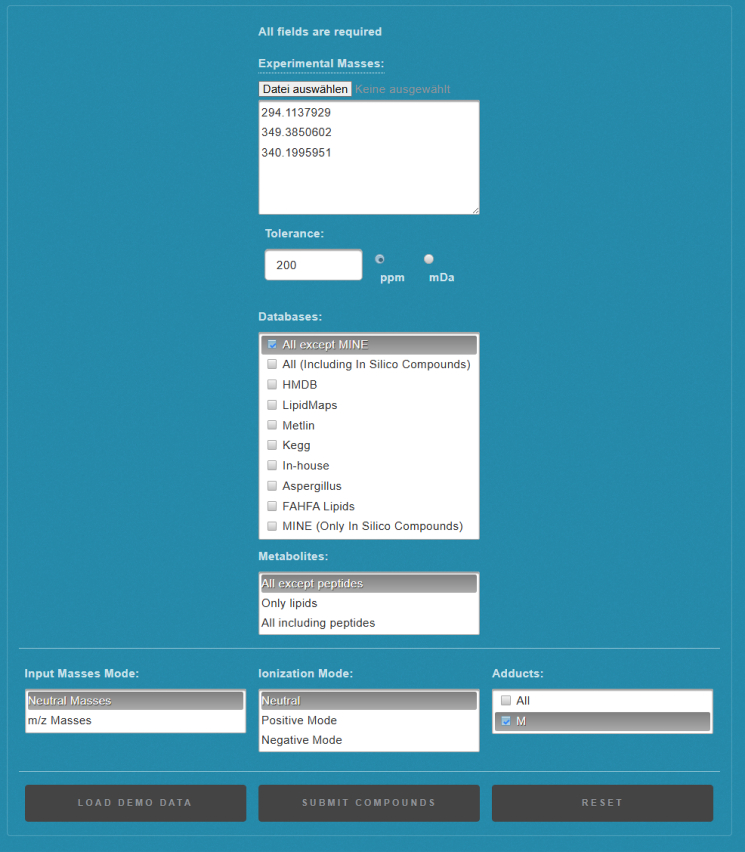
Screenshot of CEU Mass Mediator input field with required parameter settings
  e. Browse through the list of candidate compounds using the arrow keys.
  f. Download aggregated results by using the GENERATE EXCEL function at the top of the page.
    ***Note:*** CEU Mass Mediator generates an output table in the legacy Excel 97–2003 (.xls) format, which may not open smoothly in all spreadsheet programs. If compatibility issues occur, copy-paste the content into a current Excel workbook or convert the file to a modern .xlsx format before downstream processing. Each row in the CEU output corresponds to a putative compound annotation matched to one of the input masses. The table includes the queried mass, theoretical neutral mass, compound name, molecular formula, database source, and structural metadata when available. Because CEU reports all entries that fall within the specified mass tolerance, the output typically contains multiple annotations per input mass, which is expected.
    **CRITICAL:** CEU Mass Mediator performs mass-only matching and cannot distinguish structural isomers at a given mass and operates strictly within the specified mass tolerance. As a result, searches frequently return multiple plausible candidates per input mass, which is expected behavior. Do not interpret the top-ranked hit as a unique identification. Instead, retain all plausible candidates and apply additional filters depending on your experimental scope and setup before proceeding to biochemical validation.

29. Annotate candidate ligand masses using CEU Mass Mediator.
  a. Open CEU Mass Mediator https://ceumass.eps.uspceu.es/ in a web browser and select the BATCH SEARCH option under the SEARCH dropdown menu
  b. From the output file generated in Step 28 copy the list of ligand masses (Delta_Mass_Oxygen) into the “Experimental Masses” input field.
  c. Adapt the “Tolerance” and “Databases” settings according to your experimental setup and analysis scope.
    ***Note:*** In Schmidlin et al.^1^ we highlight mass-dependent error propagation effects arising from small-molecule adducts of peptides. For Orbitrap instruments operating at <5 ppm mass accuracy on peptide precursors, the effective mass error of the inferred ligand mass can be substantially higher, especially for small ligands, where error propagation may reach ∼200 ppm. Required tolerance therefore scales inversely with ligand size: small ligands require wider tolerances, whereas tolerances rapidly narrow for higher-mass ligands (Figure 4). We recommend using a global 200 ppm tolerance for CEU Mass Mediator searches and applying mass-dependent filtering to the output afterwards.
  d. Adjust the remaining search parameters as follows and submit the query using SUBMIT COMPOUNDS (Figure 5):
    i. Metabolites: All except peptides.
    ii. Input Masses Mode: Neutral Masses.
    iii. Ionization Mode: Neutral.
    iv. Adducts: M.
      ***Note:*** Once your query is complete, results will be displayed as individual result pages for each input mass.

### Shortlist candidate ligands for validation

**Timing: 4–8 weeks depending on data size**

Shortlisting refines the broad set of CEU Mass Mediator annotations into a focused group of plausible ligand candidates suitable for biochemical validation. This step integrates chemical feasibility, biological context (e.g., presence in culture medium or metabolic accessibility), and the strength and consistency of the mass spectrometric evidence supporting each Δ-mass event.

30. Shortlist candidate ligands based on chemical and biological plausibility.
  a. Combine CEU Mass Mediator output with metadata from LigandScanner (e.g., Δ-mass, reporter ion count) and group entries by input mass.
  b. Apply mass-error-propagation filtering by retaining only CEU candidates falling into the mass-dependent error window expected.
  c. Retain candidate compounds for each ligand mass, if they match basic or common MR1-binding criteria such as the presence of:
    i. An aldehyde group capable of forming a Schiff base.
    ii. An aromatic or heteroaromatic scaffold frequently observed among known MR1 ligands.
    iii. Molecular sizes consistent with known MR1-binding chemotypes.
  d. Exclude compounds that are chemically implausible in the context of the experimental design, including those that are:
    i. Not present in the culture medium.
    ii. Unrealistic in terms of cellular uptake (unless plausible in the context of intracellular conversion products).
    iii. Incompatible with the MR1 binding groove.
  e. Prioritize candidates supported by high-quality Δ-mass evidence, specifically:
    i. High reporter ion counts.
    ii. Consistent Δ-mass assignments across several spectra.
    iii. Stable retention time behavior.
  f. Manually inspect the corresponding MS/MS spectra for shortlisted candidates to confirm that the modified peptide shows a coherent fragment ion series.

***Note:*** Manual verification of MS/MS spectra and extracted ion chromatograms (XICs) is an important quality control step. Genuine MR1–ligand adducts typically show a continuous series of fragment ions consistent with the peptide sequence. In contrast, chimeric or low-quality spectra, as well as misannotated spectra are dominated by noise. The spectral quality of low-abundance ligand-modified peptides is often lower than that of the unmodified MR1 peptide.

**Figure 6:**
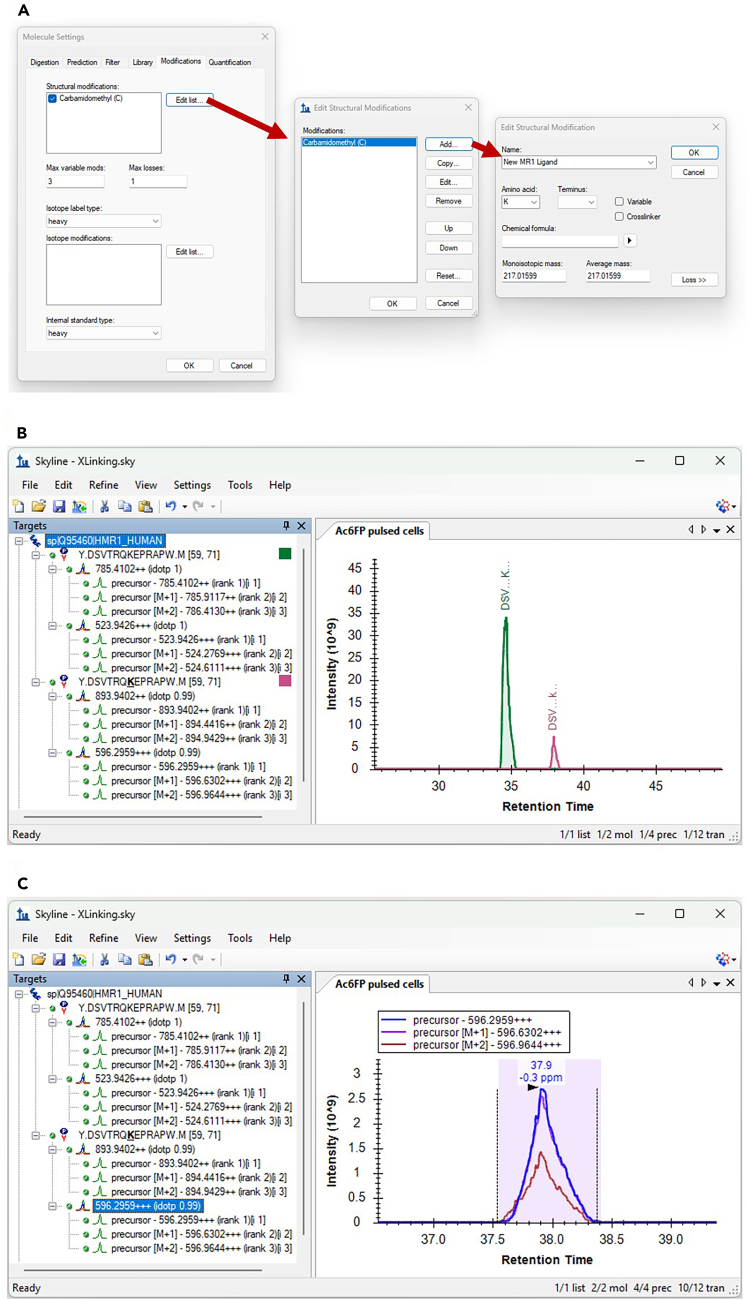
Verification of Δ-mass–defined MR1–ligand adducts using Skyline (A) Defining ligand-specific mass shifts as custom structural modifications in Skyline. Users can add a new modification under Molecule Settings → Modifications, assign it to lysine (K), and specify the inferred Δ-mass derived from LigandScanner. (B) Example Skyline view of the unmodified acetyl-6-formylpterin cross-linked MR1 peptide DSVTRQKEPRAPW as observed in a pulsing experiment, showing expected extracted ion chromatogram traces. (C) Visualization of coelution to be expected for the ions of the precursor isotopic envelope, as observed for the triply charged precursor of the acetyl-6-formylpterin modified MR1 peptide.

***Optional:*** For more stringent, orthogonal validation of candidate ligands, treat the inferred Δ-mass as a variable modification on lysine residues in a database-search engine such as PEAKS and research the raw data. This allows you to confirm that the modified peptide sequence, charge state, and fragmentation pattern are consistent with a single, site-localized modified species rather than a misassigned precursor. In parallel, define the modified MR1 peptide (unmodified sequence + Δ-mass on K43) as a custom precursor in Skyline. Visually inspect XICs of the MR1-ligand adduct and the unmodified DSVTRQKEPRAPW peptide. Strong coelution of the three most abundant isotope traces in combination with a clearly defined, sharp peak shape is indicative of a good signal. Ratios of modified to unmodified peptide provide a rough estimate of ligand occupancy (Figure 6).

**CRITICAL:** XIC-based ratios of ligand-modified to unmodified DSVTRQKEPRAPW provides a rough estimate of ligand occupancy, suitable for relative comparisons within an experiment (e.g. during protocol optimization). These ratios should not be interpreted as absolute quantitative measures across chemically distinct ligands or as a direct readout of ligand abundance of binding affinity. Ionization efficiency can differ between ligand-modified species. Accurate absolute quantification would require stable isotope–labeled ligand-modified peptide standards as internal controls. For quantitative assessment of ligand potency and biological relevance, we recommend orthogonal cell-based validation assays e.g. MR1 surface upregulation or T cell activation after ligand pulsing (see “Validation Assays”).

### Validation assays

**Timing: 3–4 days**

**Figure 7:**
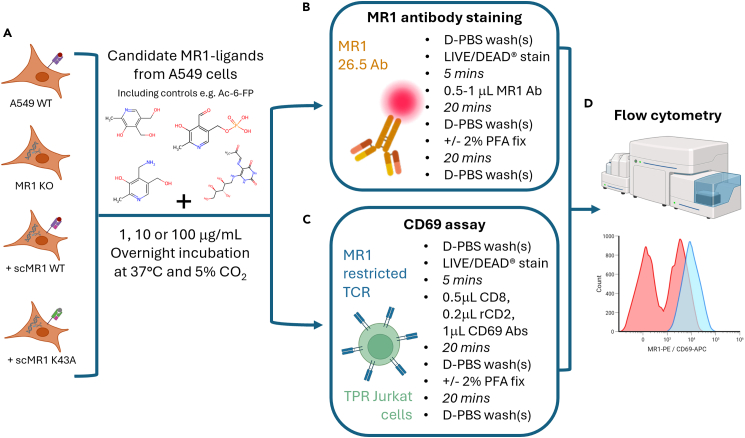
Validation assays for MR1 candidate ligands from A549 cells (A) In this step, there are 4 variations of A549 cells; WT, MR1 KO, MR1 KO + scMR1-WT and sc-MR1 K43A. Briefly as described, each cell line is treated overnight with candidate MR1-ligands identified in earlier steps of this protocol. (B and C) Working concentrations are variable to your desired conditions. After overnight incubation, cells can either (B) undergo MR1 antibody staining (C) undergo a CD69 TCR-activation assay using TPR-MC.7.G5 Jurkat cells. (D) These assays are then run on a Novocyte flow cytometer and data analysed using NovoExpress.

This section describes assays performed to test and validate candidate MR1-ligands identified in A549 cells. If the candidate MR1-ligands are binding to MR1 (and forming a Schiff base bond with K43), then MR1 cell surface expression should be upregulated on the A549 WT and A549 MR1 KO + scMR1 WT, compared to their negative counterparts (A549 MR1 KO and A549 scMR1-K43A). This upregulation is expected as MR1 traffics to the cell surface when bound via the K43 Schiff base to a ligand. Similarly, the CD69 assay is a marker for TCR activation and here the MC.7.G5 transduced into TPR Jurkat cells is used as an MR1-restricted, cancer-reactive TCR. This helps to determine whether any candidate MR1 ligands could be cancer-related and capable of activating an anticancer TCR response. See Figure 7 for an illustrative depiction of this step.

31. Antibody staining for MR1 surface expression with candidate MR1 ligands.
  a. Detach A549 WT, A549 MR1 KO, A549 MR1 KO + scMR1-WT and A549 MR1 KO + scMR1-K43A cells as described in preparation.
  b. Count the cells using a haemocytometer in a 1:1 ratio of trypan blue.
  c. Reconstitute candidate ligands shortlisted in Step 30.
    ***Note:*** Ensure the reconstitution solution is not toxic to cells at your working concentration. Serial dilutions in media may be necessary. Do not exceed a working concentration of >0.5% DMSO when adding candidate ligands to cell lines.
  d. Plate 0.1 × 10^6^ A549 cells per well in a 96 U-well plate.
    i. There should be one well for each condition (no ligand, and each subsequent candidate MR1 ligand, with replicates if desired).
  e. Add the required volume of candidate ligand compound to reach a final concentration of 100 μg/mL in 200 μL of R10 media per well.
  f. Incubate treated and untreated A549 cells for 14-16 h at 37 °C and 5% CO_2_.
  g. Wash the 96-U well plate 3 times with D-PBS as follows:
    i. Centrifuge the plate at 800 *× g* for 3 minutes.
    ii. Aspirate 150 μL medium from each well.
    iii. Add 150 μL D-PBS.
    iv. Repeat for 3 total washes.
    v. Ensure final volume per well is 50 μL after the third wash.
  h. Stain the cells with LIVE/DEAD® Fixable Violet Dead Stain Kit.
    i. Add 2 μL of a 1:40 dilution in D-PBS to each well.
    ii. Vortex at 800 rpm for 1 minute.
    iii. Incubate for 5 minutes at 20-25 °C in the dark.
  i. Add the 1 μL MR1-PE surface antibody per well.
  j. Vortex at 800 rpm for 1 minute.
  k. Incubate for 20 minutes on ice in the dark.
  l. Wash the cells 3 times as in Step 31g.
  m. Acquire samples on an ACEA NovoCyte 3005 with NovoSampler pro.
  n. Analyse the data using NovoExpress gating on:
    i. FSC-A versus SSC-A.
    ii. Single cells (FSC-A versus FSC-H).
    iii. Viable cells (marker of choice versus LIVE/DEAD® Fixable Violet Dead Stain low/negative)
  o. Plot MR1 MFI values.
    ***Optional:*** If unable to acquire the samples within 1–2 h, fix them by adding 50 μL fixing buffer, incubating for 20 min on ice in the dark then performing 3 D-PBS washes as in Step 31g. Fixed samples can be stored at 4 °C for up to 24 h.
32. CD69 assay for MR1-dependent TCR activation with candidate MR1-ligands
  a. Harvest TPR Jurkat cells (untransduced and MC.7.G5 TCR transduced) and the A549 WT and A549 MR1 KO cells as described in culture preparation.
  b. Resuspend the cells in R10 medium.
  c. Count using a haemocytometer in a 1:1 ratio of trypan blue.
  d. Plate 5 × 10^4^ TPR Jurkat cells and 1 × 10^5^ A549 target cells in 96 U-well plates, in R10.
    i. Add R10 media only to the TPR Jurkat alone controls.
    ii. Add 2 μL CD3/CD28 beads and/or 0.02 μg/mL PMA to the relevant positive control wells.
  e. Add candidate MR1-ligands at a desired working concentration (e.g., 1, 10 or 100 μg/mL).
  f. Adjust each well to a total volume 200 μL R10.
  g. Incubate for 14-16 h at 37 °C and 5% CO_2_.
  h. Wash the 96-U well plate 3 times with D-PBS as follows:
    i. Centrifuge at 800 *× g* for 3 minutes.
    ii. Aspirate 150 μL.
    iii. Add 150 μL D-PBS and repeat for 3 total washes
    iv. Final volume per well should be 50 μL.
  i. Stain with LIVE/DEAD® Fixable Violet by adding 2 μL of a 1:40 dilution in D-PBS, vortex at 800 rpm for 1 minute and incubate for 5 minutes at 20-25 °C in the dark.
  j. Add the following antibody cocktail per condition:
    i. 0.5 μL CD8-APC Vio770 (1:100)
    ii. 0.2 μL rCD2-PE (2 μg/mL)
    iii. 1 μL CD69-APC (2 μg/mL)
  k. Vortex at 800 rpm for 1 minute and incubate for 20 minutes on ice in the dark.
  l. Wash the cells 3 times as in Step 32h.
  m. Acquire samples on an ACEA NovoCyte 3005 with NovoSampler pro.
    ***Note:*** As with MR1 expression staining, samples can be fixed using fixing buffer for up to 24 h (as described in Step 31) at 4 °C if desired.
  n. Analyze the data using NovoExpress gating:
    i. TPR Jurkat population: FSC-H versus SSC-H
    ii. Single cells: FSC-A versus FSC-H
    iii. Viable cells: SSC-H versus LIVE/DEAD low/negative
    iv. TCR transduced cells: rCD2 versus CD8
    v. Generate a CD69 APC histogram and record MFI.
      ***Note:*** Necessary controls include: (i) TPR Jurkat cells alone to assess background CD69, (ii) untransduced TPR Jurkat + PMA as a positive control, and (iii) MC.7.G5 transduced TPR Jurkat + CD3/CD28 beads to activate the Jurkat cells through the TCR independently of the TCR ligand.
      ***Note:*** The CD8 antibody distinguishes TPR Jurkat cells from the A549 cells, and the rCD2 marker identifies MC.7.G5 expressing Jurkat cells.

## Expected outcomes

The expected outcome of this protocol is to detect both known and novel MR1-ligands from cell lines. Successful analyses result in proteomics data with a high quality MR1 identification characterized by high sequence coverage and IBAC values (ideally TOP 1 protein) observed for MR1 in standard quantitative proteomics database searches (e.g., PEAKS or MaxQuant). In parallel, LigandScanner provides a list of candidate ligand masses and a linked list of candidate ligand molecules generated by CEU mass mediator. User-driven data assessment together with parallel analyses in Skyline and PEAKS provides guidance for shortlisting and generates a manageable set of candidate molecules for validation.

Pulsing or co-incubation experiments with those candidate molecules are expected to result in.

(1) Increased MR1 surface expression when the molecule is a true MR1 ligand and
(2) TCR engagement in CD69 expression assays when the ligand is immunogenic, assessed using MR1-restricted TCR-transduced Jurkat cells

When working with scMR1∗01 transduced A549 cells, the protocol is expected to detect Δ-masses of pyridoxal (Δ-mass = 151.06) and PLP (Δ-mass = 231.01), as they have already been described as MR1-ligands present in these A549 cells,^1^ allowing these molecules to act as internal experimental controls.

## Quantification and statistical analysis

This protocol is not intended for absolute quantification of MR1-bound ligands. However, several semi-quantitative readouts can be obtained to support optimization, validation, and comparative analysis across experimental conditions.

### MS-based identification

Searches that include variable modifications on the MR1 K43 peptide should be performed using a 1% PSM-level FDR. For increased specificity, we recommend combining FDR control with a minimum reporter ion threshold (≥10 sequence-specific y-ions), which corresponds to the plateau region observed in our published analysis.

### Semi-quantitative MS1 assessment

Relative cross-linking efficiency or relative ligand abundance can be estimated by comparing AUC values of XICs for the ligand-linked DSVTRQKEPRAPW peptide versus the unmodified peptide. AUC values may be integrated using the top 2–3 isotopic peaks. Because ligand modification alters the peptides physicochemical properties and the relative ionization efficiency, these measurements are suitable for relative comparisons only.

### Statistical comparisons

To compare two conditions (e.g., ligand-treated vs untreated cells), unpaired two-tailed t-tests may be applied to replicate AUC values. For comparisons across multiple conditions (e.g., optimization steps or experiments with several controls), one-way ANOVA followed by Tukey’s HSD post-hoc test can be used. Statistical analysis is optional and intended to support relative comparisons, not to infer absolute ligand quantities.

## Limitations

The present protocol is conceptually limited to the detection of MR1 ligands that form Schiff base interactions with MR1 K43. Non-covalently bound ligands (e.g., diclofenac) are not stabilized during the workflow and will be lost during sample preparation. For such ligands, we recommend alternative detection strategies such as acid elution coupled with metabolomics.

In its current form, the workflow provides information exclusively on the molecular mass of the bound ligand, with accuracy dependent on ligand size. Although, in principle, MS/MS spectra could be mined for structural fragment ions, this is presently hindered by the low abundance of relevant signals. This limitation arises from the inherently low expression level of MR1, especially when compared with MHC, which currently requires the use of engineered cell lines that overexpress MR1 fused to high-specificity enrichment tags. Continued improvements in mass spectrometry sensitivity are expected to mitigate both constraints in the future.

Despite the high degree of automation in Δ-mass analysis through LigandScanner, and the availability of optional, automated orthogonal verification pipelines using PEAKS or Skyline, the final prioritization of candidate ligands still requires substantial user input. Biochemical and immunological validation remains essential for confirming ligand identity. In practice, this limits the pool of ligands that can be fully validated to those that are commercially available as pure chemical standards, such as pyridoxal and PLP in our preliminary study.^1^

## Troubleshooting

### Problem 1: Contaminations during cell culture

Related to cell culture preparation. Due to poor-sterile technique when culturing cell lines long-term or just unfortunate circumstances, there is the possibility of cell lines becoming infected by pathogens such as yeast, bacteria and mycoplasma. There is also the possibility of cross-contamination between different cell lines during culture (related to preparation Steps 1-4 and Steps 6-12 & 31-32).

### Potential solution

To resolve this problem, it is suggested to regularly check the morphology of each different cell line in culture and if unsure, compare back to morphologies found on providers such as ATCC to confirm. It should be possible to detect yeast infections visually under a microscope. It is also suggested to perform regular mycoplasma testing of any cultured cells (e.g., once a month) using the MycoAlert™ kit according to manufacturer’s instructions. If any cell lines are in question of contamination, it is always recommended to resort back to the original or an earlier passage of cells that are reliable, or re-purchasing the cells if necessary. Ensure that sterile technique is strictly followed during all cell culture steps.

### Problem 2: Low MR1 yield after Strep-Tactin enrichment

Eluted fractions show weak or undetectable scMR1 band on SDS-PAGE or Coomassie gel and/or proteomics data results in MR1 identification being of low abundance in comparison to other proteins resulting in low sequence coverage and lower overall identification scores (related to Step 15).

### Potential solution

• Verify that cells reached the required biomass (≥2.5 × 10^8^ cells) before lysis. Suboptimal growth significantly reduces total amount of MR1 recovered (related to Step 12).
• Verify that cells properly overexpress scMR1, e.g., by assessing induced surface expression with well-characterized MR1 ligands such as Acetyl-6-formylpterin or bacterial lysates, or by checking efficient killing by known MR1-restricted TCRs such as A-F7 or MC.7.G5 (related to Step 11).
• Confirm that the Strep-Tactin® XT column is fresh or properly regenerated. Exhausted resin can lead to reduced binding capacity (related to Step 14).

### Problem 3: Diagnostic MR1-derived peptide is not detected

The diagnostic MR1-derived peptide DSVTRQKEPRAPW is not detected, or is detected with insufficient signal for confident identification, indicating a failure at one or more stages of the workflow (related to Steps 24-25).

### Potential solution

• Verify complete removal of NaCNBH_3_ and TEAB: Residual reductant or buffer components can inhibit protease activity. Ensure 2 to 3 thorough buffer exchanges into PBS prior to digestion (related to Step 18).
• Avoid detergent or chaotropic carryover: Ensure no detergents, ethanol, or other inhibitory substances remain from upstream enrichment steps (related to Steps 13-23).
• Assess MR1 enrichment quality: If MR1 levels in the enriched sample are low, digestion will yield proportionally less peptide. Re-evaluate MR1 expression (related to Steps 6-11), lysis efficiency (related to Step 13), and Strep-Tactin enrichment performance (related to Step 14).
• Inspect LC-MS acquisition parameters: Ensure that MS1 and MS2 settings cover the expected m/z range and that fragmentation energy is adequate for chymotryptic peptides (related to Steps 24-25).

### Problem 4: Low or variable signal intensity of the MR1-derived peptide after digestion

The MR1-derived peptide DSVTRQKEPRAPW is detected but shows low signal intensity, poor spectral quality, or high variability across replicates (related to Steps 24-25).

### Potential solution

• Check sample pH: Chymotrypsin requires near neutral pH for optimal activity. Confirm that the sample is in PBS at pH approximately 7.4 before adding enzyme (related to Step 18).
• Confirm enzyme activity: Ensure that chymotrypsin is freshly reconstituted or taken from a freshly thawed aliquot. Loss of enzymatic activity from repeated freeze thaw cycles can markedly reduce digestion efficiency (related to Step 19).
• Adjust enzyme-to-protein ratio: If peptide coverage is low, increase the amount of chymotrypsin within the recommended range (for example toward the higher end of 1:20 w/w, related to Step 20).
• Optimize digestion time: Extend the 14-16 h incubation to up to 20 h, or perform a second chymotrypsin addition midway through the digestion (related to Step 21).

### Problem 5: LigandScanner output contains no hits with a high number of reporter ions

LigandScanner results consist predominantly of Δ-mass values supported by only a low number of reporter (fragment) ions, or entries appear empty (related to Step 28).

### Potential solution

• Ensure that score thresholds, fragment ion requirements, and ppm tolerances are not overly restrictive. Loosen tolerance values temporarily (e.g., allow higher ppm or lower fragment ion count) to determine whether the issue stems from true data quality limitations or from overly stringent settings (related to Step 27b).
• Verify MR1 enrichment quality: Poor MR1 recovery results in low-intensity ligand-modified peptide spectra and insufficient fragment ion support. Inspect the raw MS data (e.g., in PEAKS or MaxQuant). If MR1 is undetected, detected with low sequence coverage, low-scoring spectra, or only intermediate IBAC values, revisit MR1 enrichment and lysis steps (related to Steps 24-25 and Problems 2-4).
• Confirm correct centroiding during MSConvert processing: LigandScanner requires properly centroided .mgf files. Open the converted file in a text editor (e.g., Notepad++). If many m/z values occur in very close proximity (dense, non-centroided peak lists), reprocess the data using alternative centroiding settings or vendor-specific centroiding tools (related to Step 26).
• Ensure LC–MS/MS is performing to specification: Suboptimal chromatographic performance, low spray stability, or poor MS sensitivity will reduce fragment ion counts. Confirm that the system passes standard QC tests (e.g., iRT peptide mix) before re-acquiring data (related to Steps 24-25).

### Problem 6: CEU Mass Mediator output does not result in meaningful compound annotation

CEU Mass Mediator returns no high-accuracy candidate hits for the observed Δ-mass values, preventing assignment of plausible ligand candidates (related to Step 29).

### Potential solution

• Verify ppm tolerance settings: Ensure that the ppm tolerance entered in CEU Mass Mediator reflects the actual mass accuracy achieved during LC–MS/MS acquisition. If instrument performance drifted or if Δ-mass values are derived from low-intensity precursors, broaden the tolerance window accordingly (related to Step 29c).
• Consider propagated error in small Δ-mass values: Low-mass ligands (<200 Da) are disproportionately affected by MS1 error propagation. Increase the tolerance range and re-submit the Δ-mass list to evaluate whether the initial settings were too restrictive (related to Step 29c).
• Expand database search constraints: If the search was limited to predefined chemical classes or formula ranges, widen the search to include all endogenous metabolites. Novel or cell-specific ligands may not appear in narrow database filters (related to Step 29c).

## Resource availability

### Lead contact

Further information and requests for resources and reagents should be directed to and will be fulfilled by the lead contact, Thierry Schmidlin (schmidlt@uni-mainz.de).

### Technical contact

Technical questions on executing this protocol should be directed to and will be answered by the technical contacts, Hannah Thomas (thomashl9@cardiff.ac.uk) and Thierry Schmidlin (schmidlt@uni-mainz.de).

### Materials availability

Cell lines and reagents used in this study are available upon request with the appropriate material transfer agreements in place between institutes.

### Data and code availability

• Raw data are associated to the previously published article by Schmidlin et al.^1^ including all datasets analyzed during this study. They can be accessed via accompanying supplemental information (aggregated data), Passel (dataset identifier PASS05867) (LC-MS raw data), and Zenodo (data analysis codes).
• The described software LigandScanner is available for download here.
• Any additional information required to reanalyze the data reported in this protocol is available from the lead contact upon request.

## Acknowledgments

The authors would like to thank all the members of the Sewell laboratory, the Ternette laboratory, and the Schmidlin laboratory at the time of the study, as well as Dr. James Riley (University of Pennsylvania, PA, USA) for the pELNS expression plasmid. H.T. acknowledges funding from the Wellcome Trust under A.K.S., a Wellcome Investigator (220295/Z/20/Z). T.S. acknowledges funding from the Federal Ministry of Research, Technology and Space as part of the DIASyM project under grant no. 031L0218, from Enara Bio Ltd., and from the Oxford University Health Research Bridging Salary Scheme (HRBBS 0011045). The figures were partly generated using BioRender (https://biorender.com). Open access funding was enabled and organized by Projekt DEAL.

## Author contributions

Conceptualization, H.T. and T.S.; methodology, H.T., G.D., L.F.C., and T.S.; software, T.S.; investigation, H.T., G.D., L.F.C., N.T., A.K.S., and T.S.; resources, N.T., A.K.S., and T.S.; data curation, H.T. and T.S.; writing – original draft, H.T. and T.S.; writing – review and editing, all authors; visualization, H.T., G.D., and T.S.; supervision, A.K.S. and T.S.; project administration, T.S.; funding acquisition, H.T., N.T., A.K.S., and T.S.

## Declaration of interests

A.K.S. and G.D. are listed as inventors on published and pending patents (WO2023148494, EP23703861.7, and US18/793,200) pertaining to MR1-restricted recognition of cancer cells.

## References

[1] Schmidlin T., Behiry E., Thomas H., Dolton G., Marino F., Hasan S., Von Essen M., Gathungu R.M., Steigenberger B.A., Selvadurai H. (2025). MR1-ligand cross-linking identifies vitamin B6 metabolites as TCR-reactive antigens. Cell Rep. Methods.

[2] Crowther M.D., Sewell A.K. (2021). The burgeoning role of MR1-restricted T-cells in infection, cancer and autoimmune disease. Curr. Opin. Immunol..

[3] Corbett A.J., Eckle S.B.G., Birkinshaw R.W., Liu L., Patel O., Mahony J., Chen Z., Reantragoon R., Meehan B., Cao H. (2014). T-cell activation by transitory neo-antigens derived from distinct microbial pathways. Nature.

[4] Boersema P.J., Raijmakers R., Lemeer S., Mohammed S., Heck A.J.R. (2009). Multiplex peptide stable isotope dimethyl labeling for quantitative proteomics. Nat. Protoc..

[5] Holden C.A., Yuan Q., Yeudall W.A., Lebman D.A., Yang H. (2010). Surface engineering of macrophages with nanoparticles to generate a cell-nanoparticle hybrid vehicle for hypoxia-targeted drug delivery. Int. J. Nanomedicine.

[6] Borch R.F., Bernstein M.D., Durst H.D. (1971). Cyanohydridoborate anion as a selective reducing agent. J. Am. Chem. Soc..

[7] Lane C.F. (1975). Sodium Cyanoborohydride - A Highly Selective Reducing Agent for Organic Functional Groups. Synthesis.

[8] Laugel B., Lloyd A., Meermeier E.W., Crowther M.D., Connor T.R., Dolton G., Miles J.J., Burrows S.R., Gold M.C., Lewinsohn D.M., Sewell A.K. (2016). Engineering of Isogenic Cells Deficient for MR1 with a CRISPR/Cas9 Lentiviral System: Tools To Study Microbial Antigen Processing and Presentation to Human MR1-Restricted T Cells. J. Immunol..

[9] Dolton G., Thomas H., Tan L.R., Rius Rafael C., Doetsch S., Ionescu G.-A., Cardo L.F., Crowther M.D., Behiry E., Morin T. (2025). MHC-related protein 1–restricted recognition of cancer via a semi-invariant TCR-α chain. J. Clin. Investig..

[10] MacLean B., Tomazela D.M., Shulman N., Chambers M., Finney G.L., Frewen B., Kern R., Tabb D.L., Liebler D.C., MacCoss M.J. (2010). Skyline: an open source document editor for creating and analyzing targeted proteomics experiments. Bioinforma. Oxf. Engl..

[11] Kockmann T., Panse C. (2021). The rawrr R Package: Direct Access to Orbitrap Data and Beyond. J. Proteome Res..

[12] Garcia C.J., García-Villalba R., Garrido Y., Gil M.I., Tomás-Barberán F.A. (2016). Untargeted metabolomics approach using UPLC-ESI-QTOF-MS to explore the metabolome of fresh-cut iceberg lettuce. Metabolomics.

[13] Rappsilber J., Mann M., Ishihama Y. (2007). Protocol for micro-purification, enrichment, pre-fractionation and storage of peptides for proteomics using StageTips. Nat. Protoc..

